# Prolonged Cytopenia with CAR-T Cell Therapy and Management Recommendations

**DOI:** 10.46989/001c.126463

**Published:** 2025-03-26

**Authors:** Debolanle Dahunsi, Cynthia Eleanya, Akintomiwa Akintunde, Olalekan Oluwole

**Affiliations:** 1 College of Medicine University of Illinois System; 2 Center for Immuno-Oncology National Cancer Institute; 3 Medicine Meharry Medical College; 4 Vanderbilt University Medical Center

**Keywords:** Prolonged cytopenia, CAR-T, lymphoma, myeloma, lymphoblastic leukemia, Chimeric antigen receptor

## Abstract

Chimeric antigen receptor T-cell (CAR T-cell) therapy has revolutionized the treatment of lymphoid malignancies. Prolonged cytopenias, though poorly understood, have emerged as important considerations in the treatment process. In this review, we classified cytopenias into early (< 30 days post CAR T infusion), and late-occurring (after day 30 post infusion). We identified previous chemotherapy and lymphodepletion chemotherapy as the major risk factors contributing to early cytopenia. Product characteristics, such as costimulatory domains, and side effects of therapy such as cytokine release syndrome (CRS) and immune effector cell associated neurotoxicity syndrome (ICANS) were identified as contributing factors to prolonged cytopenias occurring more than 30 days post CAR-T infusion. We recommend close monitoring with frequent checks, enhanced care with granulocyte colony stimulating factor (GCSF) support for grade 3-4 neutropenia, blood transfusion for severe anemia (Hb < 7g/dL), platelets for severe thrombocytopenia (< 10,000/µL) and thrombopoietin (TPO) mimetics such as eltrombopag or romiplostim for prolonged severe thrombocytopenia in patients at high-risk of hemorrhagic complications.

## Introduction

Chimeric antigen receptor T-cell (CAR-T) therapy is rapidly becoming the standard of care in many malignancies, particularly lymphoid.^1–3^ The FDA approved CAR-T products are second generation CAR-Ts with either a CD28 or 41bbz costimulatory domain.^3,4^ Whereas these CAR-T cells are self-sufficient to effect cytotoxicity to tumors, they also retain the ability to mobilize other cells of the immune system to cause systemic inflammation.^5^ Though fludarabine and cyclophosphamide used in lymphodepletion can cause cytopenias, the expected duration is brief, and unlikely to contribute much to prolonged cytopenia extending beyond 30 days.^6^ However, many patients come into CAR-T with preexisting cytopenias, either from multiple prior lines of cytotoxic therapy or myelophthisis from tumor.^6–8^ Whereas the cytopenia from myelophthisis may resolve with clearance of tumor, marrow damage from previous cytotoxic chemotherapy may take a longer time to recover.^7,8^ Once CAR-T cells are infused into the body, they engage their target antigen, and simultaneously mobilize other cells of the immune system, together with which they produce copious quantities of IL-1, IL-6. TNF-alfa and IFN-gamma, among others.^5^ These inflammatory cytokines lead to systemic inflammation, suppression of hematopoiesis, and reduced survival of red blood cells.^5^

Since most cases of CRS resolve within the first 2 weeks of receiving CAR-T, and the previously elevated biomarkers of inflammation drop back closer to normal by day 28 - 30, previous systemic inflammation alone cannot be the cause of significant cytopenia persisting beyond 30 days.^9,10^

The impact of pancytopenia depends on the degree to which each of the cell lines are affected. Transfusion support can alleviate the symptoms of severe anemia, but severe infections can cause death in profound neutropenia, and the risk of hemorrhagic death is present in severe thrombocytopenia.

The purpose of this publication is to review the published literature, provide the pattern of cytopenias occurring in the setting of CAR-T therapy, and offer expert opinion in management.

## Methods

We searched PubMed for peer-reviewed articles that met criteria related to “chimeric antigen receptor T-cell therapy”, “DLBCL”, “MCL”, “ALL”, “Multiple Myeloma”, and “Cytopenia”. We aimed to find studies that focused on interventional clinical trials and real-world outcome publications related to adverse events such as cytopenia, including neutropenia, thrombocytopenia, and anemia. We did not limit our search by date or language, to ensure that we found the most relevant publications available. To achieve our goal, we used PubMed’s advanced search features that allowed us to refine our search for more targeted results.

Only studies that met our strict criteria were included to ensure that the information we gathered was high-quality and reliable. We then performed a comprehensive review of factors such as incidence of cytopenias, duration, and complications, which we divided into acute and chronic effects, paying close attention to time of resolution. Our expectation was that these findings will give a better understanding of the cytopenia process to the treating physicians, as well as clues to the management of cytopenias which often occur in the setting of CAR-T therapy.

For this publication, we have divided cytopenias into two groups, early (< 30 days) and late (> 30 days) post CAR-T infusion.

## Results

**Figure 1.**
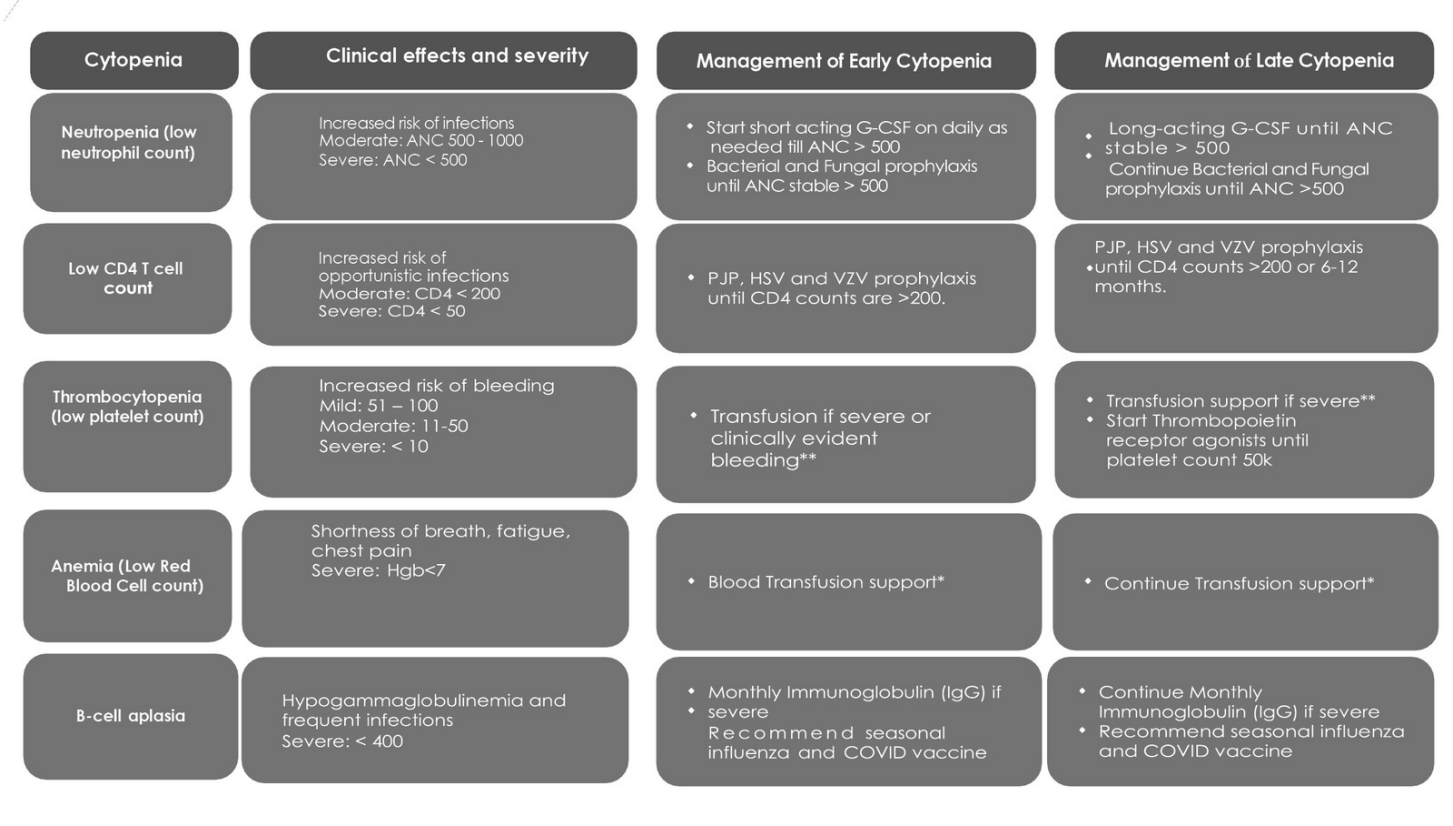
Significant Cytopenias Associated with CAR-T Therapy and Management. ANC: Absolute Neutrophil Count; G-CSF: Granulocyte-Colony Stimulating Factor; PJP: Pneumocystis Jiroveci Pneumonia; HSV: Herpes Simplex Virus; VZV (Varicella Zoster Virus); CD4: Cluster of Differentiation 4; IgG: Immunoglobulin G *Monitor for Iron Overload and or volume overload as a possible complication of Red Blood Cell Transfusion. **Monitor for Volume Overload as a possible complication of Platelet Transfusion.

Pattern of cytopenias is listed in Table 1 and schema of cytopenias in Figure 1.

**Table 1.** Cytopenias in the setting of Chimeric Antigen Receptor Therapy (CAR-T).

**Study**	**Study population**	**Sample size (N)**	**Neutropenia**		**Anemia**		**Thrombocytopenia**		**Leukopenia**		**Lymphopenia**		**Febrile Neutropenia**	
			**Any Grade**	**Grade 3 or 4**	**Any Grade**	**Grade 3 or 4**	**Any Grade**	**Grade 3 or 4**	**Any Grade**	**Grade 3 or 4**	**Any Grade**	**Grade 3 or 4**	**Any Grade**	**Grade 3 or 4**
ZUMA-1	Refractory Large B- cell Lymphoma	101	85 (84%)	79 (78%)	67 (66%)	43 (43%)	59 (58%)	38 (38%)	31 (31%)	29 (29%)	-	-	35 (35%)	31 (31%)
JULIET	Relapsed or Refractory DLBCL	111	22 (20%)	22(20%)	53 (48%)	43 (39%)	14 (13%)	13 (12%)	37 (33%)	34 (31%)	-	-	18 (16%)	17 (15%)
TRANSCEND	Chronic lymphocytic leukemia and small lymphocytic lymphoma	117	72 (62%)	71 (61%)	78 (67%)	61(52%)	58 (50%)	48 (41%)	34 (29%)	31 (26%)	24 (21%)	23 (20%)	-	-
ZUMA-2	Relapsed or Refractory Mantle-Cell Lymphoma	68	59 (87%)	58 (85%)	46 (68%)	34 (50%)	50 (74%)	35 (51%)	-	-	-	-	-	-
ELIANA	CD19+ Relapsed or Refractory B-cell ALL	75	-	3 (4%)	-	3 (4%)	-	4 (5%)	-	7 (9%)	-	-	26 (35%)	26 (35%)
KARMMA	Relapsed and Refractory Multiple Myeloma	128	117 (91%)	114 (89%)	89 (70%)	77(60%)	81 (63%)	67(52%)	54(42%)	50(39%)	35(27%)	34 (27%)	21(16%)	20(16%)
CARTITUDE	Relapsed or Refractory multiple myeloma	97	93 (96%)	92 (95%)	79 (81%	66 (68%)	77 (79%)	58 (60%)	60 (62%)	59 (61%)	51 (53%)	48 (50%)	-	-

### Early cytopenias occurring between day 1-30

Since the existing FDA approved CAR-T products are approved for second line or later use, all patients who receive CAR-T have received prior chemotherapy. Furthermore, lymphodepletion chemotherapy incorporates potent alkylating agents and nucleoside analogs; therefore, it is expected that early cytopenias will occur due to prior cytotoxic and lymphodepletion chemotherapy. Since the pattern of prior chemotherapy and lymphodepletion is variable, the occurrence of grade 3 or higher cytopenias in the period from day 1-30 is also variable. Previous cycles of chemotherapy contribute to baseline cytopenias pre-CAR-T infusion, while the depth of lymphodepletion contributes to new onset cytopenias immediately after CAR-T infusion.

Preexisting Chemotherapy: Many patients who are being evaluated for CAR-T present with cytopenias from the previous line (s) of therapy. Alkylating agents and topoisomerase inhibitors cause cytopenias during treatment and these may persist for months before it fully resolves.^11,12^ In certain instances, cytopenias may be permanent after cytotoxic chemotherapy especially in those who develop clonal hematopoiesis.^13–15^

Lymphodepletion chemotherapy: Most patients receiving CAR-T are first treated with a brief course of lymphodepletion chemotherapy.^5^ Most of the time, this consists of fludarabine and cyclophosphamide, and the dose given in combination is sufficient to produce grade 4 cytopenia in more than half of the patients treated.^1,4^ However, the period of cytopenia is expected to be brief and < 30 days.

### Late cytopenias occurring after day 30

Cytopenias persisting beyond day 30 from CAR-T infusion have a variety of etiologies.

Disease type: Patients with Acute Lymphoblastic Leukemia (ALL) tend to have a deeper and more prolonged cytopenia compared to large cell lymphoma. This difference is likely due to ALL being primarily bone marrow based and presents with baseline cytopenias pre CAR-T due to myelophthisis. Furthermore, bone marrow involvement has been identified as one of the strongest independent predictors of severe post CAR-T hematotoxicity.^16^

Costimulatory domain: Constructs that use the CD4-1BBz rather than CD28 costimulatory domains have a less prolonged and less profound cytopenia.

Occurrence of early side effects from CAR-T therapy: Patients who experience a more severe immune reactivity in the setting of CAR-T are likely to also experience prolonged cytopenias.^17^ Systemic inflammation due to CRS occurs because T and myeloid cells produce copious quantities of cytokines, including IL-1, IL-2, IFN-γ, TNF and IL-6, all of which lead to systemic inflammation and suppression of hematopoiesis^5,17^ Upon resolution of the CRS, the bone marrow function can recover. However, patients with severe and prolonged CRS may have a longer time to recover their counts.

## Predictive Models

Patients who have baseline severe cytopenias before CAR-T tend to have prolonged recovery of counts. Furthermore, elevated CRP and ferritin, which tend to track with systemic inflammation and peak CAR-T levels post infusion, signify a more inflammatory milieu which can facilitate prolonged cytopenias.

There have been attempts to predict which patients are more likely to have prolonged cytopenias in the setting of CAR-T therapy. The CAR HEMATOTOX score uses blood counts and C-reactive protein (CRP) to predict prolonged cytopenia and adverse effects.^18^ However, the data are retrospective, not stratified by growth factor utilization, and the impact of different lymphodepletion strategies may not have been captured.^18^ Though predictive and useful, we recommend not using the CAR HEMATOTOX score as the sole entity for risk stratification of patients. Rather, we suggest ensuring that all patients with baseline cytopenia before CAR-T, those who received higher lymphodepletion chemotherapy and those with significant CRS during CAR-T should be monitored more closely with daily or routine hematology laboratory tests post treatment, until toxicities have resolved or discharge from the hospital.

## Management of early cytopenia between day 1-30

We recommend a comprehensive treatment approach. This includes transfusion of red blood cells for severe anemia and platelet transfusion for severe thrombocytopenia. Expert opinion suggests transfusion for hemoglobin < 7g/dL. However, some patients may require transfusion at a higher hemoglobin level than this, due to comorbid conditions such as heart disease. Platelet transfusion is indicated for ≤ 10,000/µL. However, if there is clinical bleeding or significant platelet consumption, e.g. disseminated intravascular coagulopathy (DIC), platelet transfusions may need to be initiated at a count > 10,000/µL. When CRS is persistent, the inflammatory milieu can add to delayed platelet recovery. To manage this effectively, a combination of tocilizumab and dexamethasone is recommended for the treatment of CRS.^6,8^

Some experts, such as the European Society for Blood and Marrow Transplantation (EBMT), suggest avoiding GCSF during early days after CAR-T infusion, because of the increased risk of CRS and ICANS.^19,20^ They suggest trying to postpone its use until after day+14. However, in a small retrospective study, Galli et al. showed that there is no increase in toxicity with administering GCSF in the setting of CAR-T, especially when given as needed to mitigate the risk of neutropenic fever.^21^ The safety of administering GCSF in the case of moderate/severe CRS/ICANS is uncertain.

Although controversial, it is generally recommended to start anti-bacterial prophylaxis on day 0 or at the onset of the first occurrence of severe neutropenia, to mitigate the risk of infection. Adding antifungal prophylaxis may be a consideration in the patient who has had prolonged cytopenia before CAR-T treatment. The reason to consider prophylaxis is because many patients undergoing CAR-T have received chemotherapy and immunotherapy previously, and have diminished immune function. Furthermore, the etiology of fever post CAR-T infusion is often indistinguishable between CRS and neutropenic fever. We recommend escalating antibiotic therapy as appropriate, per institution guidelines, in the setting of neutropenic fever. We also recommend continuing prophylactic antibiotics as appropriate until resolution of fever and severe neutropenia, whichever comes later. This preventive measure should be continued until the absolute neutrophil count (ANC) has recovered to >500/µL. Additionally, antiviral prophylaxis should be initiated soon after CAR-T infusion, and continued for a period of 6 to 12 months.^6^

## Management of Late Cytopenia after Day 30

Neutropenia persisting beyond day 30 is particularly worrisome, because of the increased risk of bacterial, viral and opportunistic infections, including protozoa and fungi. When managing late cytopenia, it is important to recognize that CRS is less of a risk, hence it is reasonable to treat with GCSF to mitigate the increased infection risk that comes with prolonged neutropenia. It is also important to note that by day + 30 post CAR-T infusion, many of the patients have spent over 1 month near the CAR-T referral center and will be eager to return home. Switching to pegylated GCSF can facilitate treatment in the outpatient setting, with fewer clinic visits, but it is important to note that there are risks with prolonged pegylated GCSF use, such as inducing paradoxical hyperleukocytosis, bone pain and splenomegaly.^22,23^ In cases where there is a lack of or suboptimal responsiveness to G-CSF, a bone marrow biopsy may be necessary. CAR-T cells are often demonstrable in the marrow, indicating that cytopenia may be related to CAR-T mediated inflammation and prior therapy.^6^ If there is a high likelihood of primary disease progression/relapse, evidence of marrow fibrosis, or lineage dysplasia before CAR-T, we also consider a bone marrow aspirate for prolonged cytopenia.

When prolonged thrombocytopenia occurring in the setting of CAR-T therapy persists beyond day 30, we recommend to double check that nutritional parameters are replete, and we also recommend a TPO mimetic drug such as eltrombopag or romiplostim in those with a substantial risk of bleeding (platelets <10,000/µL) to mitigate the risk of severe hemorrhage. It is understood that the use of TPO mimetics in this instance is off label. However, there are many case series that have shown its benefit in these patients.^24^ TPO mimetics may promote hematopoietic recovery in some patients after CAR-T-cell.^6,10^

The use of hematopoietic stem cells followed by daily GCSF has been reported to aid in the recovery of prolonged cytopenias, including neutrophil, platelet, and hemoglobin levels.^16,25^

However, this intervention is only available to those multiple myeloma patients who happen to have stored cells.

Cytopenias occurring in the setting of CAR-T therapy, are an important cause of severe infections, extended hospitalization and increased mortality.^25,25–27^ Patients with severe cytopenia should be closely monitored after CAR-T therapy and treated appropriately to minimize this risk. Most CAR-T patients are treated at tertiary and academic centers, many of which have bone marrow transplant experience. These centers maintain contact with the patient in a long-term clinic and provide input in the management of complications. Growth factors and transfusion support can easily be provided by most of the CAR-T sites. However, as many community centers develop the ability to provide CAR-T treatment, more education related to management of toxicities including cytopenias is essential.

## Summary and conclusion

CAR-T has changed the paradigm of the treatment of lymphoid malignancies. However, side effects of therapy including cytopenias add to the risk of therapy and can adversely affect the quality of life of patients. Early cytopenias may occur due to pre-existing complications of previous cytotoxic therapy or lymphodepletion chemotherapy. We recommend growth factor with immediate acting GCSF and transfusion support with blood and platelets during this period, as appropriate for severe neutropenia, anemia and thrombocytopenia. There is no clear evidence that GCSF adds to toxicity during this period, so it does not need to be withheld, especially if neutropenic fever is in the list of differential diagnoses. Severe neutropenia persisting beyond day 30 occurs in a minority of patients, but it carries an increased risk of major infections, including fungal. We recommend considering pegylated GCSF in this instance, to facilitate outpatient monitoring and preserved quality of life for patients. TPO mimetics can speed up the time for platelet recovery, and stem cell boost has been used successfully in some patients with multiple myeloma who had prolonged cytopenias. Though cytopenias add morbidity and, with that, a diminished quality of life, most patients recover fully. Mortality from cytopenia post CAR-T is low, because tertiary centers are usually involved in the care of these patients. However, as more centers are now providing CAR-Ts, there is a need for focused education in the management of cytopenias to prevent morbidity and mortality

### Authors’ Contribution - CRediT

Conceptualization: Debolanle Dahunsi (Equal), Olalekan Oluwole (Equal). Data curation: Debolanle Dahunsi (Equal), Akintomiwa Akintunde (Equal). Formal Analysis: Cynthia Eleanya (Equal), Akintomiwa Akintunde (Equal), Olalekan Oluwole (Equal). Investigation: Cynthia Eleanya (Equal), Akintomiwa Akintunde (Equal), Olalekan Oluwole (Equal). Methodology: Cynthia Eleanya (Equal), Akintomiwa Akintunde (Equal). Writing – review & editing: Cynthia Eleanya (Equal), Akintomiwa Akintunde (Equal), Olalekan Oluwole (Equal). Resources: Cynthia Eleanya (Equal), Akintomiwa Akintunde (Equal). Supervision: Cynthia Eleanya (Equal), Olalekan Oluwole (Equal). Writing – original draft: Akintomiwa Akintunde (Equal), Olalekan Oluwole (Equal). Software: Olalekan Oluwole (Lead).

### Competing Interests

Debolanle Dahunsi. No competing interest

Cynthia Eleanya. No competing interest

Akintomiwa Akintunde. No competing interest

Olalekan O. Oluwole. Consultancy and advisory board for: Kite, Gilead, Janssen, ADC, Novartis, Caribou, Cargo, Nektar

### Data Availability Statement

All data are available upon reasonable request.
